# Hes1 Is Required for Appropriate Morphogenesis and Differentiation during Mouse Thyroid Gland Development

**DOI:** 10.1371/journal.pone.0016752

**Published:** 2011-02-25

**Authors:** Aurore Carre, Latif Rachdi, Elodie Tron, Bénédicte Richard, Mireille Castanet, Martin Schlumberger, Jean-Michel Bidart, Gabor Szinnai, Michel Polak

**Affiliations:** 1 INSERM U845, Université Paris-Descartes, Paris, France; 2 Department of Nuclear Medicine, Institut Gustave-Roussy, Villejuif, France; 3 Department of Clinical Biology, Institut Gustave-Roussy, Villejuif, France; 4 Paediatric Endocrinology, University Children's Hospital Basel, Basel, Switzerland; 5 Department of Biomedicine, University of Basel, Basel, Switzerland; 6 Paediatric Endocrine Unit, Necker Enfants-Malades Hospital, and Centre des Maladies Endocriniennes Rares de la Croissance, AP-HP, Paris, France; Emory University, United States of America

## Abstract

Notch signalling plays an important role in endocrine development, through its target gene Hes1. Hes1, a bHLH transcriptional repressor, influences progenitor cell proliferation and differentiation. Recently, Hes1 was shown to be expressed in the thyroid and regulate expression of the sodium iodide symporter (Nis). To investigate the role of *Hes1* for thyroid development, we studied thyroid morphology and function in mice lacking *Hes1*. During normal mouse thyroid development, Hes1 was detected from E9.5 onwards in the median anlage, and at E11.5 in the ultimobranchial bodies. *Hes1* ^−*/*−^ mouse embryos had a significantly lower number of Nkx2-1-positive progenitor cells (*p*<0.05) at E9.5 and at E11.5. Moreover, *Hes1* ^−*/*−^ mouse embryos showed a significantly smaller total thyroid surface area (−40 to −60%) compared to wild type mice at all study time points (E9.5−E16.5). In both *Hes1* ^−/−^ and wild type mouse embryos, most Nkx2-1-positive thyroid cells expressed the cell cycle inhibitor p57 at E9.5 in correlation with low proliferation index. In *Hes1* ^−/−^ mouse embryos, fusion of the median anlage with the ultimobranchial bodies was delayed by 3 days (E16.5 *vs*. E13.5 in wild type mice). After fusion of thyroid anlages, hypoplastic *Hes1* ^−/−^ thyroids revealed a significantly decreased labelling area for T4 (−78%) and calcitonin (−65%) normalized to Nkx2-1 positive cells. Decreased T4-synthesis might be due to reduced Nis labelling area (−69%). These findings suggest a dual role of Hes1 during thyroid development: first, control of the number of both thyrocyte and C-cell progenitors, via a p57-independent mechanism; second, adequate differentiation and endocrine function of thyrocytes and C-cells.

## Introduction

The thyroid gland contains two types of hormone-producing cells: thyroid follicular cells or thyrocytes, which produce thyroxin (T4) and originate mainly in the median anlage; and C-cells (parafollicular cells), which produce calcitonin (CT) and originate only in the ultimobranchial bodies.

In mice, the median anlage invaginates from the floor of the foregut starting at E8.5 and expresses *Nkx2-1*, *Pax8*, and *Foxe1*, three transcription factors whose simultaneous expression is specific of the thyroid gland [1]. From E11.5 onwards, the ultimobranchial bodies develop from the fourth pharyngeal pouch on each side [2] and express transcription factors such as *Mash1* and *Nkx2-1*. The median and lateral anlagen migrate and then fuse, at E13.5 at the definitive pretracheal position. The cells differentiate into thyrocytes expressing thyroglobulin (from E14.5) and producing T4 (from E16.5) or into C-cells expressing calcitonin (from E15.5) [3]. One factor essential for the onset of T4 synthesis is the active iodide uptake by the thyrocytes through the sodium iodide symporter (Nis) [4]. Several genes involved in thyroid development (most of which encode homeodomain proteins) have been identified, the molecular mechanisms that regulate the development and differentiation of each type of thyroid cells remains unknown.

Hes1 (hairy/enhancer of split 1) is a basic Helix-Loop-Helix (bHLH) transcriptional repressor regulated by the Notch pathway and expressed in various tissues [5]. In general, Hes1 maintains cells in the undifferentiated progenitor state by negatively regulating bHLH transcription factors such as Ngn3 in the pancreas and Mash1 in the nervous system [6]. Sound evidence exists that Hes1 is involved in the development of the pancreas [7] and pituitary gland [8] in mice. Indeed, in embryos invalidated for Hes1, the pancreas is hypoplastic. The upregulation of Ngn3 is associated with precocious and excessive differentiation [7]. Hes1 ensures proper timing of the events involved in tissue morphogenesis, as shown for neurogenesis and gliogenesis [9]. Hes1 knock-out mice die of abnormal brain development from E12 onwards [9]. Moreover, Hes1 may contribute to maintain the proliferation potential of progenitors by regulating cyclin-dependent kinase inhibitors such as p21^Cip1^, p27^Kip1^, and p57^Kip2^ [10]–[12].

Notch signalling and, more specifically, Hes1 were recently found in normal and transformed human thyrocytes, in which they regulated thyroid-specific gene expression [13]. Indeed, Hes1 appears to regulate the transactivation of the sodium iodide symporter (NIS). Moreover, decreased Notch signalling has been found in follicular and medullary thyroid cancer tissue [13], [14].

These data prompted us to investigate the role for Hes1 in thyroid development. We found that *Hes1* mutant mouse embryos had severe thyroid hypoplasia related to low thyroid progenitor cell numbers, an abnormality that was independent from p57. Moreover, the *Hes1* mutants had decreased production of both T4 and calcitonin. The decreased T4-synthesis was paralleled by a strong decrease of Nis expression. Thus, our study provides the first evidence that *Hes1* is involved in thyroid development and endocrine function of both, thyrocytes and C-cells.

## Results

To investigate the role for *Hes1* in thyroid development, we measured *Hes1* expression during thyroid differentiation and we studied thyroid development in mice lacking *Hes1*.

### Hes1 is expressed during thyroid murine development

**Figure 1:**
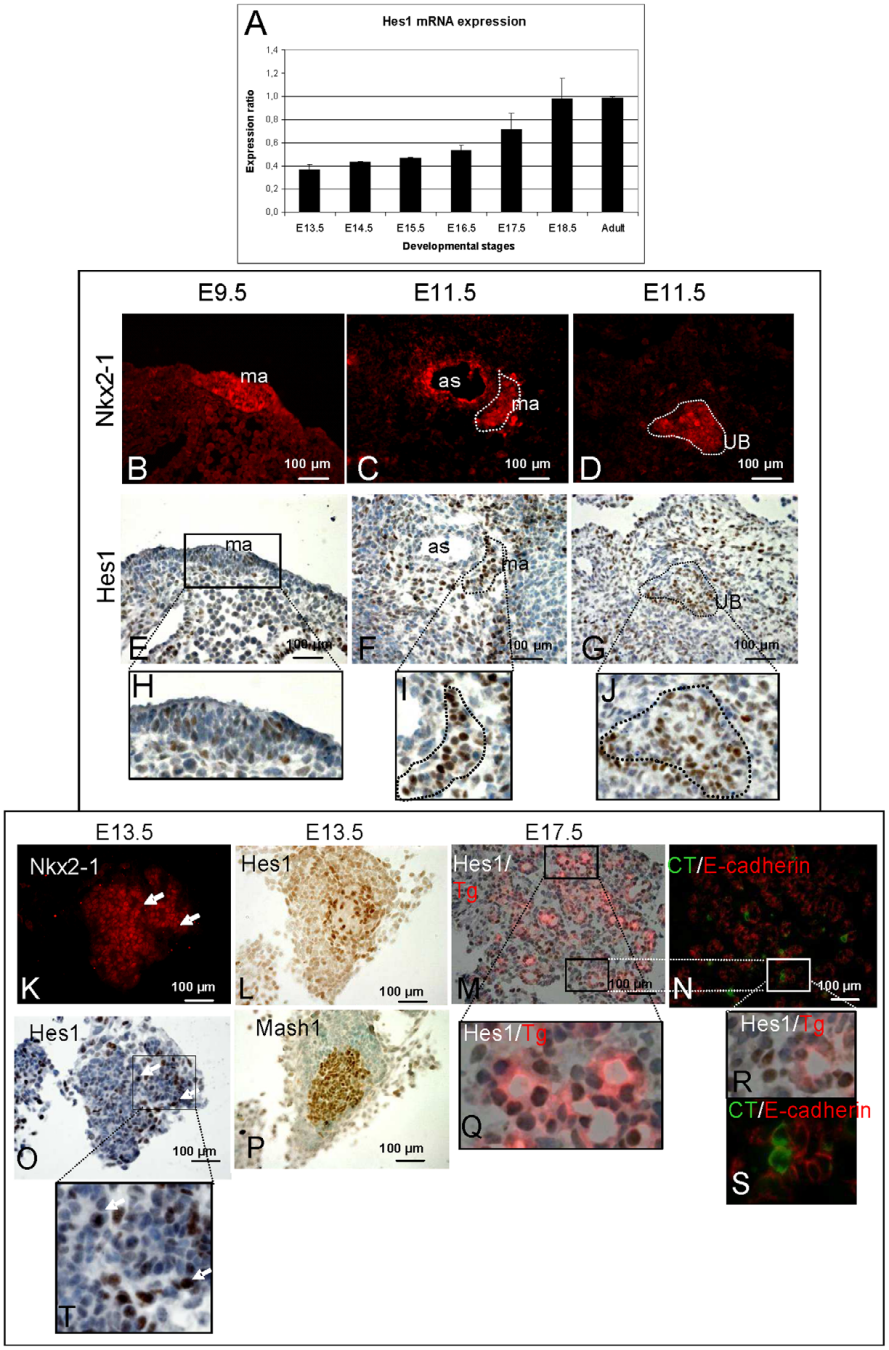
Hes1 expression during thyroid development. A: RT-QPCR analysis of *Hes1* mRNA expression in embryonic and adult thyroids.*Hes1* expression increased significantly from E13.5 until E18.5 (p<0.05). B-T: Immunohistochemistry for Hes1 and Nkx2-1 in whole embryos at E9.5 and E11.5 (sagittal sections) (B–J). At E9.5, Nkx2-1 stained the median thyroid anlage evaginating of the endoderm. At E11.5, Nkx2-1 stained the median anlage along the aortic sac. At E11.5, Nkx2-1 marked the symmetrical ultimobranchial bodies from the endoderm of the fourth pharyngeal pouch. Hes1 co-localised with Nkx2-1 cells in the median anlage and in the ultimobranchial bodies. At E13.5, immunohistochemistry for Hes1, Nkx2-1 and Mash1 is shown in dissected thyroids at E13.5 (K, L, O, P, S). In dissected thyroids at E17.5 (M, N, Q, R) Hes1/TG, and CT/E-cadherin co-labelling was analysed in adjacent sections. H, I, J, Q, R, S represent enlarged areas. Hes1 is expressed in progenitors of and in mature thyrocytes and C-cells. ma: median anlage; UB: ultimobranchial body, as: aortic sac.

*Hes1* mRNA expression was significantly increased in thyroid glands from E13.5 to E18.5 embryos (*p<0.05* E13.5 vs. E18.5) (**Figure 1A**). Then, we studied expression of Hes1 on mRNA and protein level by use of in situ hybridization (ISH) and of a specific antibody on wild-type and *Hes1−/−* embryos (Figure S1). Hes1 protein was only expressed in a subset of Nkx2-1-positive precursors of the median anlage at E9.5, (**Figure 1B, E, H**) but was clearly expressed at E11.5 in both, the median anlage lining the aortic sac (**Figure 1C, F, I**) and in the ultimobranchial bodies in a lateral position (**Figure 1D, G, J**). Further, Hes1 positive cells were also present in the thyroid anlagen surrounding tissues (**Figure 1E, F, G**). To determine which cell types expressed Hes1, we investigated co-expression of Hes1 with antibodies against Nkx2-1, thyroglobulin (TG), calcitonin (CT), and Mash1. At E13.5, Hes1 was expressed in some Nkx2-1-positive cells (**Figure 1K, O, S**). From E15.5 to E18.5 and in adult glands, Hes1 showed nuclear staining in most Nkx2-1-positive (*data not shown*) and TG-producing thyrocytes within thyroid follicles (**Figure 1M, N, Q**). Hes1 was expressed at E13.5 in Mash1-positive cells (**Figure 1L, P**) and from E15.5 onwards in CT-producing C-cells (**Figure 1R**). This Hes1 expression profile in the developing as well as adult thyroid tissue suggested a role of Hes1 for differentiation and endocrine function of Nkx2-1-positive thyrocytes precursors as well as for Nkx2-1/Mash1-double-positive C-cell progenitors.

### Thyroid hypoplasia in *Hes1* mutant embryos

**Figure 2:**
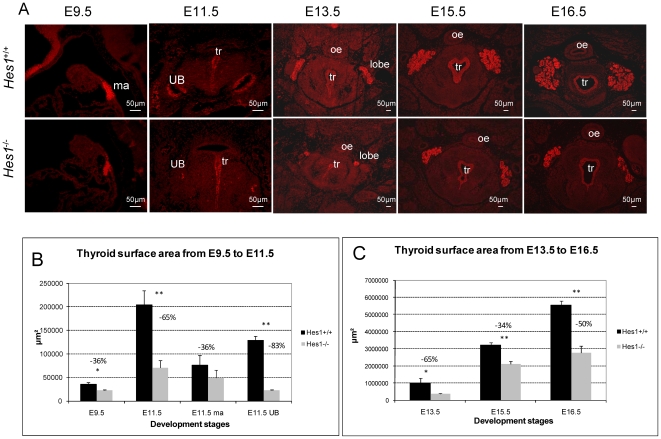
Thyroid hypoplasia in *Hes1^−/−^* embryos. A: Nkx2-1 staining at E9.5 (sagittal sections) and at E11.5, E13.5, E15.5, and E16.5 (transverse sections) in littermates. Total thyroid surface area (µm^2^) was quantified using Nkx2-1 staining at each embryonic stage, B: The results represent the surface area of three embryos per stage and per genotype. UB: ultimobranchial body; ma: median anlage; tr: trachea, oe: oesophagus. Student's test, * *p*<0.05 and ** *p*<0.01.

To determine the role for Hes1 in thyroid development, we investigated mice lacking functional *Hes1* alleles. This well-characterized null allele lacks the first 3 exons of the Hes1 gene, which encodes the bHLH domain of Hes1 [15]. Hes1 null embryos die during fetal development, allowing examination of the morphology of the thyroid in wild-type and Hes1 mutants at the onset of endocrine function at E16.5 but not beyond. Nkx2-1 is expressed in undifferentiated thyrocytes as well as C-cells precursors in the median anlage and the ultimobranchial bodies from E9.5 onwards and remains expressed in both cell types after onset of endocrine function. Thus, we evaluated the size of the developing thyroid by measuring the surface area of Nkx2-1-positive cells at E9.5, E11.5, E13.5, E15.5 and E16.5. We compared at each stage three *Hes1^−/−^* and three wild type embryos (**Figure 2A, B and C**). At E9.5, we performed sagittal sections of embryos and we located the Nkx2-1 positive thyroid median anlage within the endoderm covering the tongue in analogy with previous work [1], [3] (**Figure 2A**). From E11.5 to E16.5, we performed transverse sections of embryos and we located the Nkx2-1 positive thyroid lobes in the typical lateral position on both sides of the trachea and larynx, and the isthmus connecting the two lobes by crossing the trachea in a pretracheal position (**Figure 2A**). In accordance with the general Nkx2-1 expression pattern, the tracheal epithelium showed also specific positive staining. At E9.5, the median anlage of *Hes1* mutants was already significantly smaller than in wild type mice, the Nkx2-1-positive surface area being 36% smaller (p<0.05) (**Figure 2B** **)**. At E11.5, the overall Nkx2-1-positive surface area of both thyroid anlages in *Hes1^−/−^* mice was 65% (p<0.01) smaller than in the wild type mice (median anlage: −36%; ultimobranchial bodies: −83%) (**Figure 2B** **)**. At E13.5, E15.5, and E16.5, the thyroids of *Hes1^−/−^* mice were 65% (p<0.05), 34% (p<0.01), and 50% (p<0.01) smaller, respectively, than those of wild type mice (**Figure 2C**). Thyroid size of heterozygous *Hes1^+/−^* thyroids was normal compared to wild-type littermates, but was significantly bigger than *Hes1^−/−^* thyroids (p<0.01) (*data not shown*). This significant reduction of thyroid size at all stages was not paralleled by a general hypotrophy or reduction of the overall body size of Hes1*^−^* ^/*−*^ mutants.

These results suggest that *Hes1* expression in the developing thyroid was required for the gland to reach its normal size. Thyroid anlagen specification, in contrast, was normal in *Hes1^−/−^* mice.

### Thyroid shape and fusion abnormalities in *Hes1^−/−^* mice

**Figure 3:**
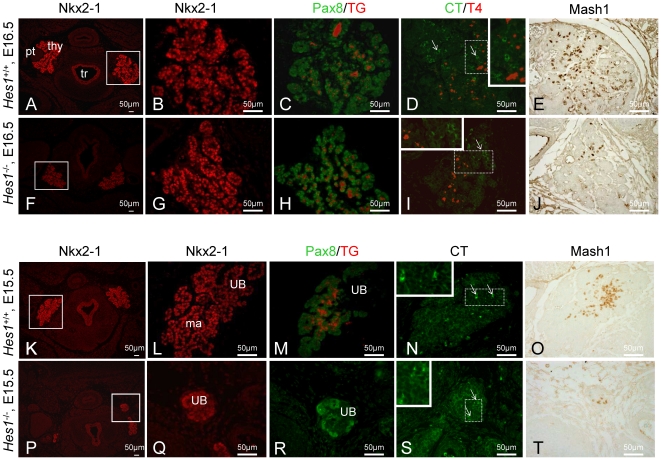
Thyroid morphology and expression of thyrocyte and C-cell markers at late embryonic stages. Staining for Nkx2-1 (A, B, F, G, K, L, P, Q), Pax8 and TG (C, H, M, R), CT and T4 (D, I, N, S), and Mash1 (E, J, O, T) at E16.5 (A–J) and E15.5 (K–T) in transverse sections from wild type and *Hes1^−/−^* mice. Boxed areas were enlarged (B–E, G–J, L–O, Q–T). Thyroid fusion was delayed by 3 days in *Hes1^−^* ^/*−*^ thyroids compared to wild type thyroids. tr: trachea; thy: thyroid; ma: median anlage; pt: parathyroid; UB: ultimobranchial body.

The wild type thyroid showed a high level of organisation at E16.5: Nkx2-1 was expressed in all thyroid cells, Pax8 in TG-producing thyrocytes organized in small thyroid follicles, and CT and Mash1 in C-cells scattered throughout the gland in a parafollicular position (**Figure 3A**–**E**). Mash1 expression was maintained in the developing thyroid, a finding at variance with results by Kameda et al. [16]. The *Hes1^−/−^* thyroid at E16.5 contained Nkx2-1-, Pax8-, TG-, and T4-expressing cells scattered throughout the gland (**Figure 3F**–**I**). Unexpectedly, we found that CT- and Mash1-expressing cells were clustered in a specific region of the gland (**Figure 3I**–**J**). We sought an explanation to this difference with the wild type gland by examining thyroids at two earlier stages, E15.5 (**Figure 3K**–**T**) and E13.5 (*data not shown*). At E15.5, fusion of the ultimobranchial bodies with the median anlage was apparent in wild type mice but not in *Hes1^−/−^* mice (n = 3) (**Figure 3K, L, P, Q**). The E15.5 ultimobranchial bodies of the *Hes1^−/−^* mice expressed a moderate number of Pax8-positive cells and few CT- and Mash1-positive cells (**Figure 3R, S, T**). Thus, absence of *Hes1* was associated with a 3-day delay in the fusion of the ultimobranchial bodies to the median anlage: fusion occurred at E16.5 in the *Hes1^−/−^* mice and at E13.5 in the wild type mice. Moreover, CT- and Mash1-expressing cells in *Hes1^−/−^* thyroids had not spread throughout the gland at E16.5 due to delayed fusion of the ultimobranchial bodies with the median anlage. At this stage, CT- and Mash1-expressing cells were localized at the cranial part of the thyroid lobes.

Additional morphological abnormalities such as abnormalities of the isthmus are further detailed in Table 1 for each stage. We found no abnormalities in the expression of E-cadherin, a protein known to be involved in cell-cell adhesion (*data not shown*) [17].

**Table 1:** Morphological abnormalities in *Hes1^−/−^* mutant thyroid.

Embryonic stage	Thyroid anlages	Structural features of the thyroid gland in *Hes^−/−^* mice	Decrease in surface area (%) *vs* WT
E9.5		Elliptical, close to aortic sac, as in WT	36%
E11.5	MA	1out of 3 median anlage separated laterally	65%
	UB	1 out of 3 embryos: UB lacking on one side	36%
	total		83%
E13.5		1 out of 3 thyroids: no isthmus3 out of 3 thyroids: UB not fused with MA	65%
E15.5		2 out of 3 thyroids: enlarged isthmus3 out of 3 thyroids: UB not fused with MA	34%
E16.5		2 out of 3 thyroids: enlarged isthmusUB fused with MA, but C-cells not disseminated in thyroid gland	50%

MA, median anlage; UB, ultimobranchial bodies; WT, wild type.

These findings indicated that *Hes1* was required to ensure normal shape, and size. However, formation of thyroid follicles was normal in *Hes1^−/−^* thyroids.

### Decreased CT, Nis and T4 surface area in the *Hes1^−/−^* thyroid

**Figure 4:**
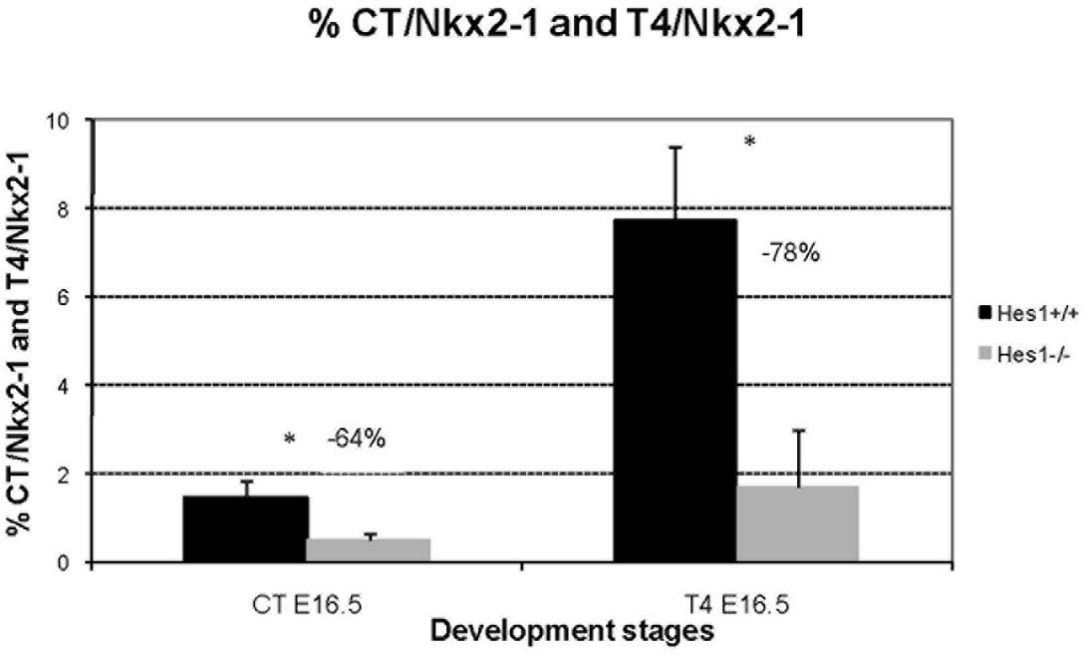
Decreased CT and T4 expression in *Hes1* ^−/−^ thyroids. CT and T4 surface areas were quantified at E16.5 in wild type and *Hes1^−/−^* mice and compared to the Nkx2-1-positive total thyroid surface area. Three embryos per genotype were used for quantification.Chi-square test, * *p*<0.05.

To investigate onset of thyroid function in *Hes1^−/−^* mice, we determined the endocrine signature of the thyroid cells by measuring CT and T4 immunoreactivity. The CT-positive surface area relative to the Nkx2-1-positive surface area was smaller than in wild type mice, by 64% at E16.5 (*p*<0.05) (**Figure 4** **,** **Figure 3D, I**). Moreover, the T4-positive surface area normalized to the Nkx2-1 positive surface area at E16.5 was 78% lower in *Hes1^−/−^* mice than in wild type mice (*p*<0.05). Total thyroid surface area (Nkx2-1-positive surface area) in *Hes1^−/−^* thyroids was lower by 50% at E16.5, compared to wild type thyroids. The decreases in CT- and T4-positive surface areas were more pronounced than the decrease in total thyroid surface area.

These data indicate that the hypoplastic thyroids in *Hes1^−/−^* animals show also intrinsically decreased endocrine function at E16.5.

**Figure 5:**
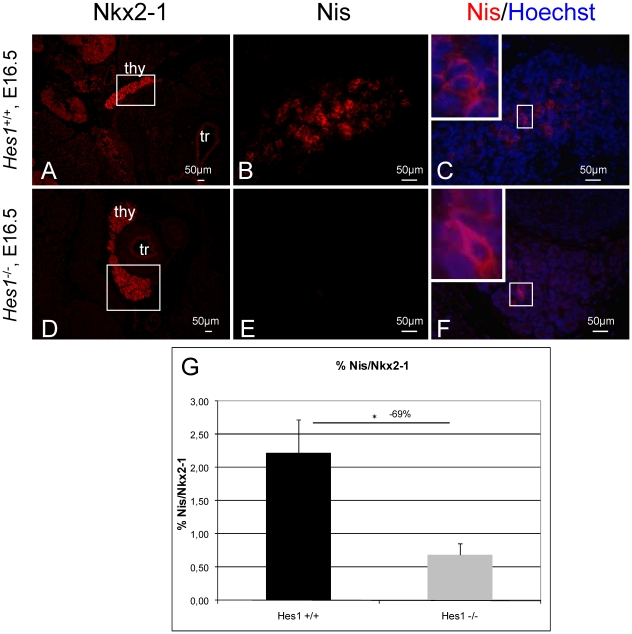
Nis protein expression and quantification in *Hes1^−/−^* thyroids. The Nkx2-1 staining in A and D localizes the thyroid gland. In B, C, E, F, Nis protein labelled thyrocytes. In C and F, co-staining of Nis and Hoechst visualizes Nis in the basolateral membrane of the thyrocytes and Hoechst in the nucleus of the thyrocytes. In G, Nis surface areas were quantified at E16.5 in wild type and *Hes1^−/−^* mice and compared to the Nkx2-1-positive total thyroid surface area. Three embryos per genotype were used for quantification.Chi-square test, * *p*<0.05.

Ferretti et al. have shown that Nis was an apparent Hes1 target gene. Indeed, HES1 transactivated NIS directly in human adult thyrocytes [13]. Nis promotes the active iodide uptake by the thyrocytes, which is a crucial step for the onset of thyroid hormone synthesis and is expressed from E15.5 onwards in mice [18]. Thus, we quantified the Nis-positive surface area in relation to the total Nkx2-1 positive surface area in *Hes1^−/−^ vs*. wild type mice. The ratio of Nis-positive to Nkx2-1 positive surface area at E16.5 was 69% lower in *Hes1^−/−^* mice than in wild type mice (*p*<0.05) (**Figure 5A**–**G**) reflecting a reduced iodide uptake capacity.

### Thyroid hypoplasia in *Hes1^−^* ^/*−*^ mutants due to decreased progenitor number

**Figure 6:**
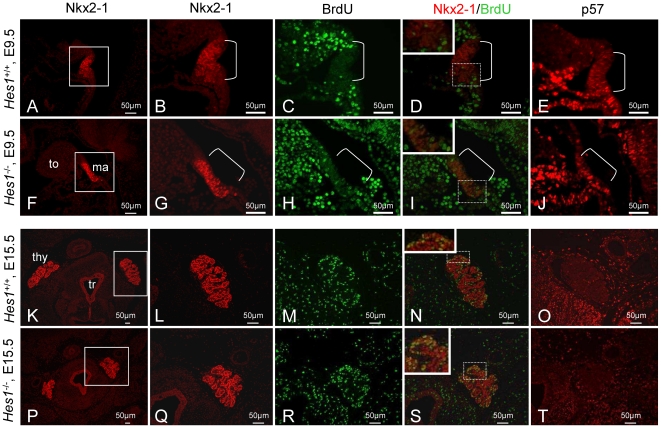
Cell proliferation and p57 expression in thyroids at E9.5 and E15.5. Immunohistochemistry of Nkx2-1 (A, B, F, G, K, L, P, Q), BrdU (C, H, M, R), and p57 (E, J, O, T) in transverse sections from wild type mice (A–E and K–O) and *Hes1^−^* ^/*−*^ mice (F–J and P–T) at E9.5 (A–J) and E15.5 (K–T). Boxed areas were enlarged (B–E, G–J, L–O, Q–T). tr: trachea; thy: thyroid; ma: median anlage; to: tongue.

**Figure 7:**
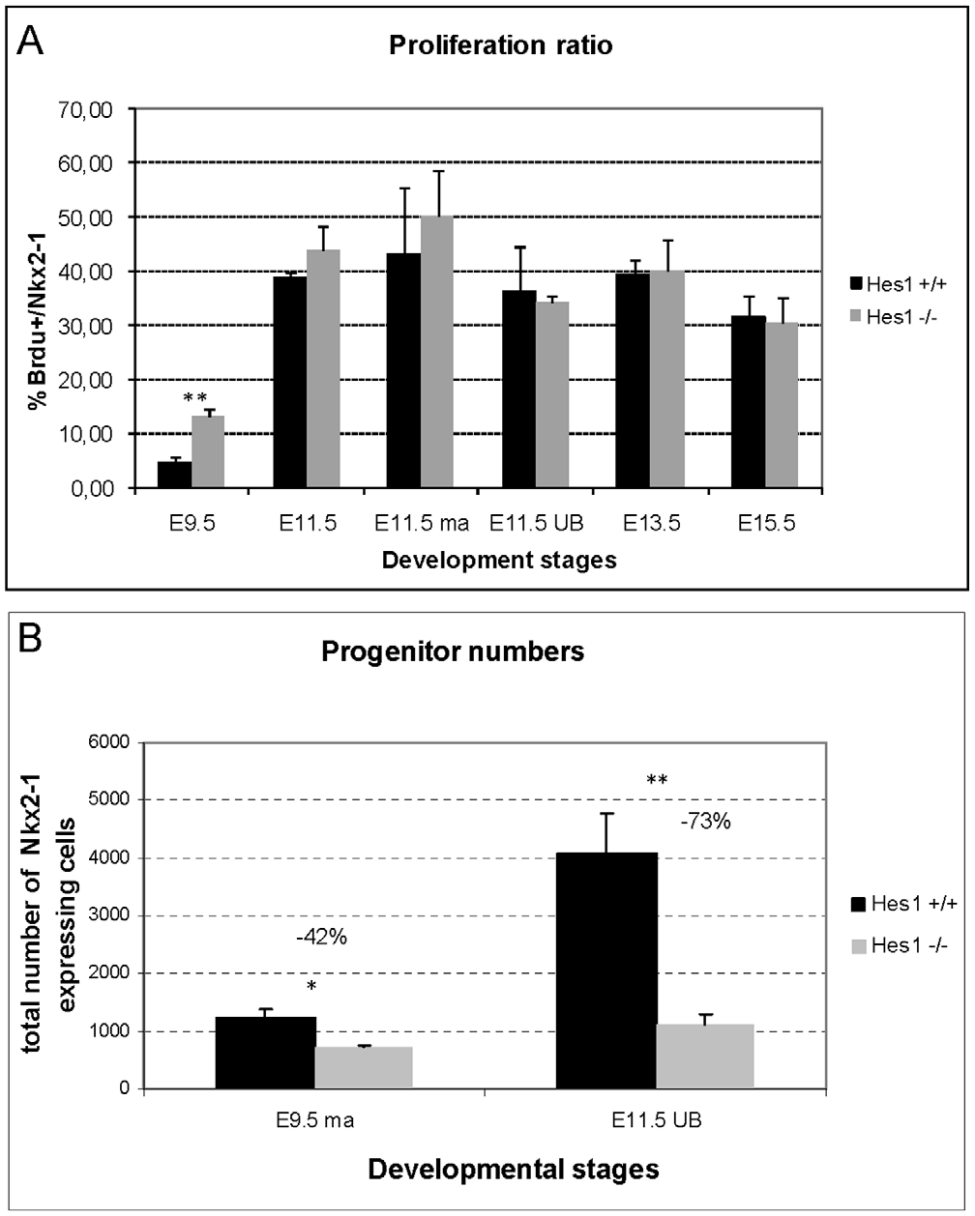
Proliferation ratio and progenitor cell number. A: The proliferation ratio was calculated as the proportion of Nkx2-1-positive cells labelled with BrdU at E9.5, E11.5, E13.5, and E15.5 in wild type and *Hes1^−^* ^/*−*^ thyroids. At E11.5, before fusion the median thyroid anlage (ma) and the ultimobranchial bodies (UB) were quantified separately. Three embryos per stage and per genotype were used for quantification. Chi-square test, ** *p*<0.01. B: Number of progenitor cells at E9.5 in the median thyroid anlage (ma) and at E11.5 in the ultimobranchial bodies (UB). Three embryos per stage and per genotype were used for quantification.Student's test, * *p*<0.05 and ** *p*<0.01.

We investigated four possible hypotheses for thyroid hypoplasia in *Hes1^−/−^* thyroids: 1) decreased proportion of proliferating cells in the developing thyroid, 2) increased proportion of apoptotic cells, 3) accelerated cell differentiation, and 4) decreased pool of progenitor cells from specification. To assess proliferation, we determined the ratio of BrdU-labelled Nkx2-1-positive cells over all Nkx2-1-positive cells. At E9.5, the proportion of Nkx2-1-positive cells labelled by BrdU was 4.6% in wild type thyroids (**Figure 6A**–**D** and **Figure 7A**) and 13.2% (*p*<0.001) in *Hes1^−/−^* thyroids (**Figure 6F**–**I** and **Figure 7A**). At E11.5, E13.5, and E15.5, this ratio was not significantly different between *Hes1^−/−^* and wild type thyroids (**Figure 6K**–**N, P–S** and **Figure 7A**). Neither did we find any evidence supporting a role for increased apoptosis in the thyroid hypoplasia (*data not shown*). *Hes1^−/−^* and wild type thyroids were not different regarding the time of appearance of TG and T4 immunostaining in thyrocytes or of CT immunostaining in C-cells. Thus, hormone-producing cells did not differentiate earlier in *Hes1^−/−^* than in wild type mice, in keeping with data on pancreas development [7]. In contrast, the number of Nkx2-1 positive progenitor cells was significantly lower in *Hes1^−/−^* thyroids than in wild type thyroids: at E9.5 in the median anlage by 42% (p<0.05) and at E11.5 in the ultimobranchial bodies by 73% (p<0.01), respectively (**Figure 7B** **).**

To investigate the cause of the progenitor cell depletion, we analysed the expression of cyclin-kinase inhibitors (CKIs). CKIs play a major role in regulating cell cycle exit and have links with Notch signalling [10]. We found that the CKI p57 was clearly expressed in the median anlage in both *Hes1^−/−^* and wild type mice at E9.5 (**Figure 6E, J**) but not at the other stages studied (from E11.5 to E16.5) (**Figure 6O, T**). These data were consistent with the low proliferation ratio at E9.5 in the wild type thyroid: 4.6% (**Figure 6A**–**D** and **Figure 7A**) while the rate of proliferation was on average 40% from E11.5 onwards. Thus, p57 was not involved in the thyroid progenitor cell deficiency in *Hes1^−/−^*. Moreover, we also analysed expression of two further CKIs. However, we did not find differences in the expression of p21 and p27 in Hes1 wild type *vs.* Hes1*^−^* ^/*−*^ mice (*data not shown*).

Therefore, neither accelerated differentiation, increased apoptosis, or decreased proliferation, provided an explanation for thyroid hypoplasia. Further, the determination of the number of progenitors of the thyroid primordium by Hes1 was independent of p57, p21 and p27.

## Discussion

**Figure 8:**
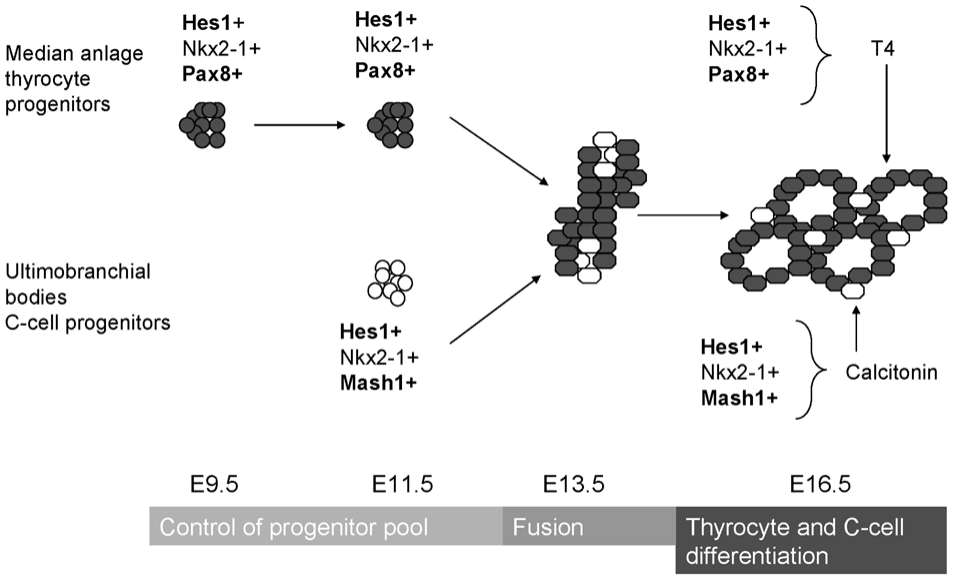
Proposed roles of Hes1 during murine thyroid development and function. Hes1 controls the number of thyrocyte progenitors in the median anlage (in grey) at E9.5 and C-cell progenitors in the ultimobranchial bodies (in white) at E11.5. Hes1 has a role for the correct fusion of the two anlages at E13.5. At E16.5, Hes1 is involved in normal endocrine function in differentiated thyrocytes and C-cells.

Hes1 is well known in development of different tissues, particularly endoderm derived endocrine organs like pancreas, and neuroendocrine cells of lung. Hes1 controls normal development by regulating proliferation and differentiation and so cell fate. In mutant *Hes1* mouse embryos, we found that the thyroid gland was hypoplastic and hypofunctional. Thus, the thyroid gland is not the first example of an endodermic tissue affected by Hes1. This study allowed us to involve a new gene in thyroid development (**Figure 8**). However, specification of the median anlage and the ultimobranchial bodies was independent of Hes1.

### Hes1 expression in the thyroid gland

Nuclear Hes1 staining occurred in both thyroid anlages as early as they appeared. This expression pattern on the protein level was maintained throughout development as well as in adult tissues. Hes1 expression was heterogenous at early embryonic stages (E9.5 and E11.5) in the median anlage, as observed by others during early pancreas development (E10–11) (*data not published*). From E13.5 onwards we found an increasing expression of Hes1 on mRNA level with highest expression levels in the adult tissue. The maintained Hes1 expression during differentiation of thyrocytes and C-cells as well as in the mature tissue differs from decreasing Hes1 expression in endocrine pancreas development [19].

### Thyroid hypoplasia and delayed fusion of median and lateral thyroid anlages in *Hes1^−/−^* mutants

Hypoplasia of the thyroid anlagen in *Hes1^−/−^* mice was already present as early as E9.5 in the median anlage and E11.5 in the ultimobranchial bodies. The ratio in size reduction between mutant and wild type thyroids remained constant between E9.5 and E16.5.

Further analysis revealed that the number of progenitors was clearly decreased in the median anlage and more so in the ultimobranchial bodies. This correlates with the more abundant expression of Hes1 in the ultimobranchial bodies at E11.5 rather than in median anlage at E9.5. This observation is in accordance with data showing that the number of embryonic progenitor cells is a limiting factor for pancreatic size [20]. Thus, Hes1 influences the number of thyroid progenitor cells of thyrocytes as well as C-cells available in both thyroid anlages, most likely by a cell-autonomous way.

CKI play a major role in regulating cell cycle exit and have links with Notch signalling [10]. In the pancreas, lack of effect of Hes1 on p57, a member of the CKI family, caused decrease of pancreatic progenitors and resulted in accelerated differentiation [7], [10]. To characterize the mechanism leading to reduced progenitor pool in the *Hes1^−/−^* thyroid, we compared the expression of p57 in mutant and wild-type tissues. At E9.5 cell proliferation was limited and the cells were in cycle arrest due to strong p57 expression in the median anlage in *Hes1^−/−^* and wild-type mice. In contrast, at E11.5, in the median anlage as well as in the ultimobranchial bodies, the cells proliferated actively, and very few cells expressed p57. The fact, that p57 expression was not different in mutant and wild type thyroids from E9.5 onwards suggests that Hes1 regulates the progenitor pool in a p57 independent manner.

Previous work has already described a weak progenitor cell proliferation in the wild-type median anlage as it evolves through the budding stage [3]. These authors proposed recruitment of new progenitors from neighbouring Nkx2-1 negative endoderm as possible mechanism for normal bud growth. On the other hand, Lania et al. showed recently, that Tbx1 regulates, via Fgf8, the size of the early thyroid primordium by regulating the proliferation of endoderm derived thyrocytes precursors [21].

Other causes of hypoplasia such as modification of the onset of differentiation or increased apoptosis within the *Hes1^−/−^* thyroids were excluded. Further, proliferation ratio did not differ in mutant and wild type mice from E11.5 onwards, in accordance with constant size reduction ratio between thyroids of mutants *vs*. wild type until E16.5. In contrast to what could have been expected, proliferation ratio at E9.5 was 2.8 times higher in the median anlage of *Hes1^−/−^* mutants than in wild type thyroids. This might reflect a compensatory mechanism to produce a normal number of thyroid progenitor cells. However, this compensation has not reached the normal number of progenitor cells leading to hypoplasia of the thyroid. In summary, the mechanism of thyroid hypoplasia remains open and is different to what is decribed during pancreas development.

In *Hes1^−/−^* mice, Jensen et al described disturbed fusion of the hypoplastic dorsal and ventral pancreatic evaginations at E16 [7]. In wild type mice, the median anlage reaches the definitive paratracheal position at E12.5 and the ultimobranchial bodies originating from the fourth pharyngeal pouch fuse with the upper pole of the symetrical thyroid lobes at E13. Interestingly, and in accordance with defective fusion of the ventral and dorsal pancreas, we found a three day delay of the fusion of the hypoplastic thyroid anlages in Hes1 mutant mice. The complete fusion occurred only at E16.5. Hes1 plays an important role for correct organogenesis of the thyroid gland.

### Reduced endocrine thyroid function in *Hes1^−^* ^/*−*^ mutants

Onset of T4 synthesis occurs at E16.5 in mice, as soon as structural and functional differentiation is completed. Structural differentiation comprises thyroid follicle formation, the functional unit of the thyroid gland. In contrast to general hypoplasia and fusion abnormalities, follicle formation was normal in both, wild type and mutant mice. However, the ratio of T4/Nkx2-1 positive surface area was strongly reduced in *Hes1* mutant mice, suggesting reduced endocrine function of thyrocytes lacking *Hes1*. Hes1 has recently been shown to directly transactivate the promoter of Nis. In *Hes1^−/−^* mice, Nis/Nkx2-1 ratio was also clearly reduced. Thus, reduced Nis-expression with consecutive decreased iodide accumulation could provide a possible explanation for decreased endocrine function of thyrocytes.

On the other hand, C-cell differentiation depends on *Mash1* expression. According to Kameda et al, *Mash1* expression in the thyroid is present at a high level at E13.5 and absent from E14.5 onwards, which allows C-cell differentiation [16]. In neuronal and lung tissues, *Mash1* and *Hes1* expressions were inversely correlated [22], [23]. Therefore, we expected to see an increase in Mash1 expression in *Hes1^−/−^* thyroids. However, in variance to published data, Mash1 expression was maintained until E16.5 in wild type thyroids [16]. In contrast, *Hes1^−/−^* thyroids showed very few Mash1-expressing cells, and a strongly reduced ratio of CT/Nkx2-1 positive surface area, suggesting a reduced endocrine function of C-cells. Then, we can not conclude that the expression of Hes1 and Mash1 show an inverse correlation in mouse thyroid development.

It was shown in *Hoxa-3* and *Eya1* mutant mice that the fusion of the thyroid anlagen plays a crucial role for normal differentiation [24], [25]. Disordered differentiation is only observed in *Eya1* knock-out mice after fusion of the ultimobranchial bodies expressing Eya1 and the median anlage which does not express Eya1 [25]. Thus, the delayed fusion in *Hes1^−/−^* mice provides a possible explanation for impaired endocrine function.

T4 production and, thyroid size depend on TSH/TSH-receptor signalling in differentiated thyrocytes. A marked reduction in TSH production has been reported in the pituitary of *Hes1^−/−^* mice [8]. As TSH is secreted from E14.5 by the pituitary gland and TSH-receptor protein is expressed in the thyroid from E14.5 onwards, thyroid hypoplasia noted before E14.5 in *Hes1^−/−^* mice cannot be ascribed to TSH/TSH-receptor activity [26]. However, we cannot exclude an additional role of TSH deficiency for decreased T4 production seen at E16.5 in the *Hes1^−/−^* mice.

These data allowed us to postulate that Hes1 involved in the thyroid differentiation of the thyrocytes and the C cells.

### *Hes1* function in the developing and mature thyroid

We found that the expression of both *Hes1* mRNA and Hes1 protein increased during development and persisted in the adult mouse thyroid. The Notch pathway has been shown to be involved in human thyroid function and in thyroid carcinomas arising from thyrocytes as well as C-cells [13], [14]. Ferretti et al. found that *Hes1* directly activated the expression of sodium iodide symporter [13], crucial for the onset of thyroid function [4]. In our study, T4-producing cells were found in *Hes1^−/−^* thyroids. Therefore, *Hes1* is one potential transcriptional co-factor for NIS-expression but seems not to be indispensable for T4 synthesis. The maintained expression of Hes1 in the adult thyroid suggests nevertheless an important role for thyroid function. These results are in contrast to pancreas development: during early pancreas development, Hes1 downregulates a key transcription factor of endocrine cells named Ngn3 [19].

Additional studies are needed to elucidate the role for Hes1 in maintaining thyroid function.

In conclusion, our data provide for the first time evidence that Hes1 is an additional regulator of thyroid development and differentiation (**Figure 8**), beside the known thyroid enriched transcription factors such as Nkx2-1, Foxe1, and Pax8: Hes1 maintains an adequate pool of thyroid progenitor cells via a p57-independent mechanism, determining normal thyroid size. Moreover, Hes1 is necessary for normal endocrine function of both, thyrocytes and C-cells in the differentiated thyroid gland.

## Materials and Methods

### Animals

*Hes1^−/−^* mice were previously generated by replacing the first three *Hes1* exons, including the bHLH domain, with a neomycin-resistance cassette [15]. CD1-Hes1 mice were maintained under specific pathogen-free conditions at the Bomholtgaard breeding centre (Ry, Denmark). Genotyping was performed as previously described [7]. Embryos at specific ages were obtained from *Hes1^+/−^* females mated with *Hes1^+/−^* males. *Hes1^−^* ^/*−*^ embryos die just before or at birth. Thyroid morphology was examined in wild type and *Hes1^−^* ^/*−*^ embryos. Given the variability of phenotype penetrance in the nervous system and pancreas [7], [15], we chose *Hes1^−^* ^/*−*^ embryos whose visual nervous system was abnormal. All experiments were conducted in accordance with the principles and procedures outlined in the NIH Guide to the Care and Use of Experimental Animals and with approval from the Danish regulatory authorities use of experimental animals (document 2006/561-1125) and approval from Hagedorn Research Institute.

For bromodeoxyuridine (BrdU) experiments, BrdU (20 mg/kg) was injected intraperitoneally to each pregnant mouse 1 h before sacrifice.

### Dissection

Embryos were removed from pregnant mice at different days of gestation. Briefly, in a first step, the proximal part of the trachea, the larynx and the thyroid were visualized under a dissecting loupe (Leica, Leitz DMRB, Reuil-Malmaison, France) and in a second step, the thyroid glands were micro-dissected in analogy to the procedure used in adult mice. Only bilobed tissues with intact isthmus were assigned as thyroids and used for further experiments.

### Real-Time PCR

To determine the *Hes1* transcript level in thyroid rudiments from E13.5 to E18.5 and in adult thyroid, real-time PCR was performed using the 7300 Fast real-time PCR system (Applied Biosystem, Paris, France). Total RNA was isolated using the Qiagen RNeasy Microkit (Qiagen, Courtaboeuf, France) and treated with DNase I to eliminate genomic contamination. We used 2 to 5 thyroid rudiments for RNA extraction depending on embryonic stages. The Superscript Invitrogen protocol (Invitrogen, Carlsbad, CA) was used for reverse transcription of 100 ng of each RNA sample. The synthesized cDNA was diluted to 1/20, and 5 µl was used per PCR reaction. Each reaction consisted of Taqman® universal PCR master mix (Applied Biosystems, Carlsbad, CA), primers, and a Taqman-labelled probe specific for each gene (Applied Biosystems, Carlsbad, CA). Each reaction was run using the universal thermal cycling protocol (95°C for 10 min followed by 40 cycles at 95°C for 15 s and 65°C for 1 min). Peptidylpropyl isomerase A was used as an endogenous control. Adult thyroid cDNA was used as a calibrator sample. The data were analysed using the comparative cycle threshold method and presented as the fold change in gene expression, normalized for a calibrator whose value equalled 1.

### Histological analyses

After sacrifice of pregnant mice, whole embryos or dissected thyroids were fixed in 3.7% formalin, and embedded in paraffin. Sections (4 µm thick) were collected and processed for immunohistochemistry (sagittal sections at E9.5 and E11.5; and transverse sections at E13.5, E15.5, and E16.5). The antibodies were used at the following dilutions: rabbit anti-Hes1 (1/500, gift from Dr T. Sudo), rabbit anti-Nkx2-1 (1/2500, Biopat, Milan, Italy), rabbit anti-Pax8 (1/2000, Biopat, Milan, Italy), rabbit anti-Mash1 (1/20, Becton-Dickinson, Franklin Lakes, NJ), mouse anti-TG (1/100, Dako, Glostrup, Denmark), rabbit anti-calcitonin (1/400, Dako, Glostrup, Denmark), mouse anti-T4 (1/10000, AbD Serotec, Raleigh, NC), rabbit anti-Nis (1/200, gift from Thierry Pourcher), mouse anti–E-cadherin (1/100, Becton-Dickinson, Franklin Lakes, NJ), rabbit anti-p57 (1/10, Abcam, Cambridge, UK) and mouse anti-BrdU (1/4, Amersham, Fairfield, CT). The fluorescent secondary antibodies used were Alexa Fluor 488 goat anti-rabbit and anti-mouse and Alexa Fluor 633 goat anti-rabbit and anti-mouse antibodies (1/400, Alexa, Invitrogen, Carlsbad, CA).

The paraffin-embedded sections were deparaffinised by serial passages in toluene and in alcohol. The sections were then heated for 15 minutes in Antigen Retrieval solution (Biogenex, San Ramon, CA) in a microwave, cooled, blocked with TBS-BSA 3%-Triton 0.3%, and incubated with the primary antibody overnight at 4°C. After three 5-minutes washes in TBS-Tween 0.1%, the sections were incubated with the appropriate fluorescent secondary antibody for 2 hours at room temperature then mounted with mounting liquid. Photographs were taken using a fluorescence microscope (Leitz DMRB; Leica, Nussloch, Germany) and digitised using a Hamamatsu C5810 chilled 3CCD camera (Middlesex, NJ). For Mash1, the detection protocol was adapted from the Dako kit protocol for peroxidase-labelling amplification (Dako, Glostrup, Denmark).

For Hes1 antibody, a different protocol was used: tissues were fixed at 4°C in 4% paraformaldehyde in PBS, cryoprotected in 15% sucrose-PBS at 4%°C overnight, embedded in 15% sucrose−7.5% gelatine in PBS, and frozen at −50°C in isopentane. Cryosections 4 µm in thickness were prepared. Endogenous peroxidase activity was quenched by incubation in 0.3% hydrogen peroxide in methanol for 30 minutes. The sections were microwaved in EDTA solution, cooled, and incubated with blocking solution (TBS-BSA 3%-Tween 0.1%) then with the primary antibody for 1 h at 37°C. Then, the sections were incubated with biotinylated secondary antibody for 1 hour. The next step was immunostaining using the Vectastain ABC kit (Vector Laboratories, Burlingame, CA) according to the manufacturer's instructions. The sections were then incubated in 3,3′-diaminobenzidine tetrahydrochloride and counterstained with hemalun-eosin. Specificty of Hes1 antibody was tested on Hes1^−/−^ embryos. No Hes1 staining was detected in Hes1*^−^* ^/*−*^, while positive Hes1 staining was reproducibly detected on wild-type tissues (Figure S1).

### Quantification

Photographs were taken using a fluorescence microscope (Leitz DMRB; Leica) and digitalised using a chilled 3CCD camera (C5810; Hamamatsu). To measure the surface area labelled with Nkx2-1, CT, Nis, or T4, immunohistochemistry was performed on every second section from each embryo throughout the whole tissue. The sections were then analysed using ImageJ 1.32 s (public domain software, www.rsbweb.nih.gov/ij). The Nkx2-1-positive surface areas per section were summed to obtain the total surface area per thyroid, in µm^2^. The surface areas positive for CT, Nis and T4, markers of late thyroid differentiation, were normalized for the Nkx2-1 surface area representing the total thyroid. At least three thyroids were analysed per condition. The results are reported as mean±SEM. In each tissue analysed with ImageJ 1.32 s, representative sections were also analyzed manually by two different persons to validate fluorescent surface area measurements.

TUNEL experiments were done using an *in situ* cell death detection kit (Roche, Neuilly-sur-Seine, France) according to the manufacturer's instructions. Nkx2-1 immunostaining was then performed. To determine the percentage of apoptotic thyroid cells, we counted the frequency of TUNEL-positive cells among 500 Nkx2-1-positive cells.

Proliferation of Nkx2-1-positive cells was estimated by counting BrdU-positive nuclei among Nkx2-1-positive cells on every second section throughout the complete tissue. The chi-square test was used to determine whether differences were statistically significant.

For all tests and for all stages, we systematically analyzed three *Hes1^−/−^* and three wild type embryos.

## Supporting Information

Figure S1 **Expression of Hes1 by ISH and by IHC (immunohistochemistry) on wild-type and** ***Hes1*** **−** ***/*** **− embryos.** A: Hes1 ISH and Nkx2-1 IHC on sagittal sections of wild-type and *Hes1*−*/*− embryos at E10.5 stage. B: Hes1 IHC and Nkx2-1 IHC on sagittal sections of wild-type and *Hes1*−*/*− embryos at E10.5 stage. No staining for Hes1 by ISH and IHC in Hes1−/− embryos in the Nkx2-1 positive thyroid anlage, while clear and reproducible staining by HIS and IHC was found in wild-type embryos.
(TIF)
