# Supplementary figures and images for: Hes1 Is Required for Appropriate Morphogenesis and Differentiation during Mouse Thyroid Gland Development
Source: PLoS One. 2011 Feb 25;6(2):e16752. doi: 10.1371/journal.pone.0016752 (PMC3045378; doi:10.1371/journal.pone.0016752)

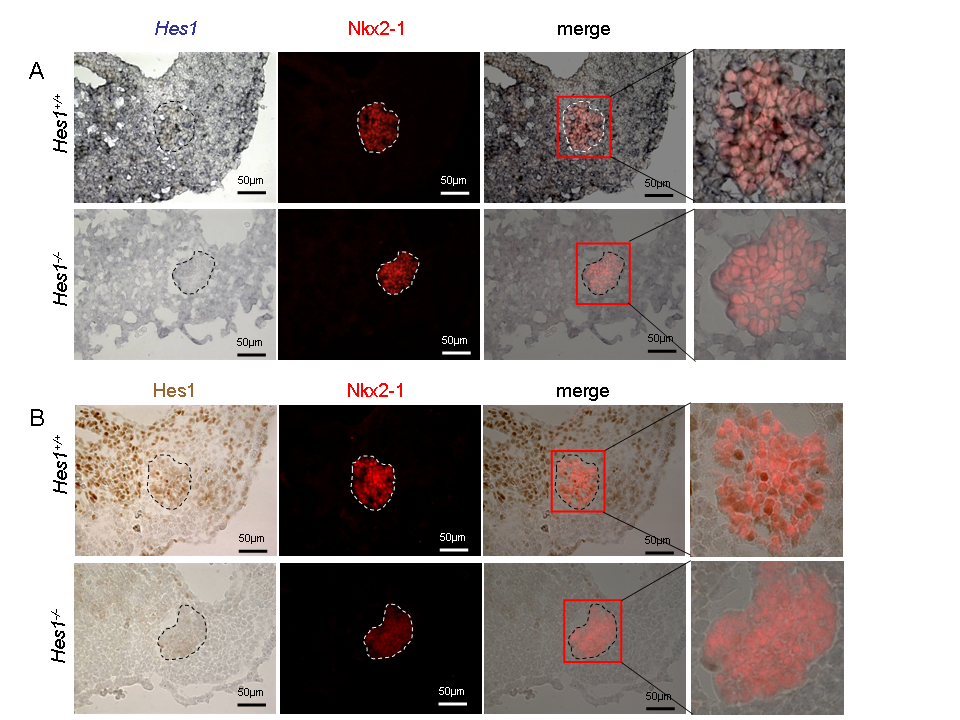

Supplement: Figure S1 — Expression of Hes1 by ISH and by IHC (immunohistochemistry) on wild-type and Hes1 − / − embryos. A: Hes1 ISH and Nkx2-1 IHC on sagittal sections of wild-type and Hes1−/− embryos at E10.5 stage. B: Hes1 IHC and Nkx2-1 IHC on sagittal sections of wild-type and Hes1−/− embryos at E10.5 stage. No staining for Hes1 by ISH and IHC in Hes1−/− embryos in the Nkx2-1 positive thyroid anlage, while clear and reproducible staining by HIS and IHC was found in wild-type embryos. (TIF) [file pone.0016752.s001.tif]
